# Transcriptional Regulators of Ecdysteroid Biosynthetic Enzymes and Their Roles in Insect Development

**DOI:** 10.3389/fphys.2022.823418

**Published:** 2022-02-08

**Authors:** Takumi Kamiyama, Ryusuke Niwa

**Affiliations:** ^1^College of Biological Sciences, Graduate School of Life and Environmental Sciences, University of Tsukuba, Tsukuba, Japan; ^2^Life Science Center for Survival Dynamics, Tsukuba Advanced Research Alliance (TARA), University of Tsukuba, Tsukuba, Japan

**Keywords:** ecdysteroids, ecdysone, steroidogenic enzyme, transcription factors (TFs), *Drosophila*, Halloween genes

## Abstract

Steroid hormones are responsible for coordinating many aspects of biological processes in most multicellular organisms, including insects. Ecdysteroid, the principal insect steroid hormone, is biosynthesized from dietary cholesterol or plant sterols. In the last 20 years, a number of ecdysteroidogenic enzymes, including Noppera-bo, Neverland, Shroud, Spook/Spookier, Cyp6t3, Phantom, Disembodied, Shadow, and Shade, have been identified and characterized in molecular genetic studies using the fruit fly *Drosophila melanogaster*. These enzymes are encoded by genes collectively called the Halloween genes. The transcriptional regulatory network, governed by multiple regulators of transcription, chromatin remodeling, and endoreplication, has been shown to be essential for the spatiotemporal expression control of Halloween genes in *D. melanogaster.* In this review, we summarize the latest information on transcriptional regulators that are crucial for controlling the expression of ecdysteroid biosynthetic enzymes and their roles in insect development.

## Introduction

Steroid hormones are bioactive small molecules that induce many physiological events in both vertebrates and invertebrates. Steroid hormones are received by their specific nuclear receptors and activate downstream gene transcription. In arthropods, including insects, the principal steroid hormones, ecdysteroids, including the most biologically active 20-hydroxyecdysone (20E), play essential roles in their development, such as embryogenesis, molting, metamorphosis, oogenesis, diapause, immune response, stress resistance, seasonal plasticity, learning, memory acquisition, and longevity (Ishimoto and Kitamoto, 2011; Uryu et al., 2015; Belles, 2020; Yamanaka and Okamoto, 2020; Kamiyama and Niwa, 2021a,b; Kurogi et al., 2021; Nunes et al., 2021; van der Burg and Reed, 2021).

**FIGURE 1:**
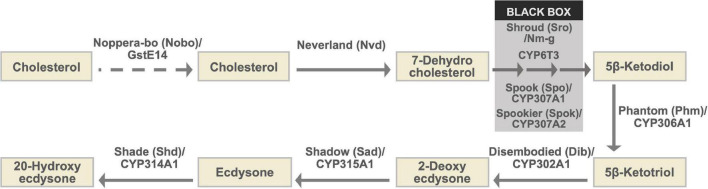
The ecdysteroid biosynthesis pathway and Halloween enzymes. As the conversion steps between 7-dehydrocholesterol and 5β-ketodiol have not been chemically characterized, they are collectively called the “Black Box.” Notably, *spo* and *spok* are paralogous to each other (Ono et al., 2006; Komura-Kawa et al., 2015). Although genetic and physiological analyses have suggested that Nobo plays a role in cholesterol intake or trafficking in the PG, endogenous substrates of Nobo have not been identified. Therefore, the role of Nobo is indicated by the dashed arrow in this illustration.

During larval and pupal development, ecdysteroids are biosynthesized from dietary cholesterol and plant sterols in a special endocrine organ called the prothoracic gland (PG) (Figure 1). Studies during the 2000s and early 2010s successfully identified and characterized a number of essential ecdysteroid biosynthetic enzymes (Niwa and Niwa, 2014a). These enzyme-encoding genes are collectively called the Halloween genes (Gilbert, 2004). After their discovery, it was found that the levels of expression of Halloween genes in the PG correlated well with the levels of hemolymph ecdysteroid titers. This finding was reminiscent of the expression of mammalian steroidogenic genes, which reflects the production of steroid hormones (Mizutani et al., 2015). In mammals, the strict control of the expression of steroidogenic genes is regulated by several key steroidogenic transcription factors (TFs), including Ad4BP/steroidogenic factor 1 (SF-1) and liver receptor homolog-1 (Parker et al., 2002). By contrast, elucidation of ecdysteroidogenic TFs and their regulatory roles in insects has lagged far behind that of mammalian steroidogenic transcriptional regulation.

Here, we describe the recent findings regarding the transcriptional regulation and chromatin remodeling in the expression of Halloween genes. Particularly, we aim to update our knowledge on the role of ecdysteroidogenic TFs in the control of ecdysteroid biosynthesis in the PG during development.

## Ecdysteroid Biosynthetic Enzymes Encoded by Halloween Genes

Some of the genes encoding the ecdysteroidogenic enzymes were first identified in a series of *Drosohila melanogaster* classical genetic mutants that caused embryonic lethality and a defect in cuticle structure characterized by naked and polished cuticles (Chávez et al., 2000; Gilbert, 2004). Because naked cuticle embryos were superficially characterized, these mutants were referred to as ghosts and specters, including *disembodied* (*dib*), *noppera-bo* (*nobo*), *phantom* (*phm*), *shadow* (*sad*), *shade* (*shd*), *spook* (*spo*), and *shroud* (*sro*) (Chávez et al., 2000; Enya et al., 2014). *D. melanogaster* embryonic lethal mutants showing the naked cuticle phenotype and defects related to ecdysteroid biosynthesis were designated and classified as “Halloween mutants” (Gilbert, 2004). In addition, the genes responsible for Halloween mutants and their products were named “Halloween genes” and “Halloween enzymes,” respectively. However, embryonic lethal phenotypes identical to Halloween mutants have not been reported for a part of ecdysteroid biosynthetic enzymes (Niwa and Niwa, 2014a). Despite that, researchers usually collectively utilize the term “Halloween genes” and “Halloween enzymes” to refer to all identified ecdysteroid biosynthetic genes and enzymes, respectively. In this review article, we follow this customary scientific terminology.

The ecdysteroid biosynthetic pathway and ecdysteroid biosynthetic enzymes encoded by Halloween genes are illustrated in Figure 1 and Table 1 (Gilbert et al., 2002; Niwa and Niwa, 2014a; Kamiyama and Niwa, 2021b, and references therein). All Halloween enzymes, except for Shd, act as catalytic enzymes in the PG. Endogenous substrates of Neverland (Nvd), Phm, Dib, Sad, and Shd have been identified (Figure 1 and Table 1), while the precise biochemical roles of other enzymes have not been elucidated. Nevertheless, Sro, Spo, Spok, and CYP6T3 are thought to function in the “Black Box” because the larval arrest phenotype of knockdown of *sro*, *spok*, or *Cyp6t3* can be rescued by oral administration of 5β-ketodiol, but not 7-dehydrocholesterol (Figure 1; Ono et al., 2006; Niwa et al., 2010; Ou et al., 2011). After release from the PG to the hemolymph, ecdysone is converted to 20E by Shd in the peripheral tissues (Figure 1).

**TABLE 1:** A list of ecdysteroid synthetic enzymes encoded by Halloween genes.

Enzyme name	Synonym	CG number in *D. melanogaster*	Enzyme type	Substrate	Orthologs outside *Drosophilidae*^e^	Requirement in embryogenesis^f^	References^g^
Noppera-bo (Nobo)	GstE14	CG4688	GST^a^	Unknown	YES	YES	Enya et al., 2014
Neverland (Nvd)	–	CG40050	Rieske	Cholesterol Lathosterol^c^	YES	NO	Yoshiyama et al., 2006; Lang et al., 2012
Spook (Spo)	CYP307A1	CG10594	P450	Unknown^d^	YES	YES	Namiki et al., 2005; Ono et al., 2006
Spookier (Spok)	CYP307A2	CG41624	P450	Unknown^d^	NO	NO?	Ono et al., 2006
Shroud (Sro)	–	CG12068	SDR^b^	Unknown^d^	YES	YES	Niwa et al., 2010
CYP6T3	CYP6T3	CG8457	P450	Unknown^d^	NO	?	Ou et al., 2011
Phantom (Phm)	CYP306A1	CG6578	P450	5β-Ketodiol	YES	YES	Niwa et al., 2004; Warren et al., 2004
Disembodied (Dib)	CYP302A1	CG12028	P450	5β-Ketotriol	YES	YES	Chávez et al., 2000; Warren et al., 2002
Shadow (Sad)	CYP315A1	CG14728	P450	2-Deoxyecdysone	YES	YES	Warren et al., 2002
Shade (Shd)	CYP314A1	CG13478	P450	Ecdysone	YES	YES	Petryk et al., 2003

*^a^GST, Glutathione S transferase.*

*^b^SDR, short-chain dehydrogenase/reductase.*

*^c^Drosophila pachea Neverland catalyzes conversion from lathosterol, but not cholesterol, to 7-dehydrocholesterol.*

*^d^The substrate should be an intermediate between 7-dehydrochlesterol and 5β-Ketodiol.*

*^e^“YES” means that orthologs have been found in the genome of organism other than Drosophilidae species.*

*^f^“YES” means that mutant embryo of that gene shows Halloween mutant phenotype because of ecdysteroid deficient.*

*^g^First identification of mutant phenotype and/or biochemical function of each enzyme.*

Among the Halloween enzymes, Nobo, also known as GstE14 in *D. melanogaster* and GSTE7 in the silkworm *Bombyx mori*, is the most recently identified one known to contribute to ecdysteroid biosynthesis. Nobo belongs to the family of glutathione *S*-transferase (GST). Therefore, we briefly introduce Nobo in this review article. *Nobo* is predominantly expressed in the embryonic PG precursor, larval PG, and adult ovarian follicle cells (Chanut-Delalande et al., 2014; Enya et al., 2014, 2015). *D. melanogaster* genetic null mutants of *nobo* show embryonic lethality and typical cuticular patterning defects, which are identical to the phenotypes of other Halloween embryonic mutants (Chávez et al., 2000). In addition, the larval-arrest phenotype of PG-specific RNAi against *nobo* was almost fully rescued by oral administration of 20E or cholesterol. As cholesterol is thought to be the most upstream precursor, it was hypothesized that Nobo does not convert ecdysteroidal intermediates, but rather plays a crucial role in cholesterol intake and transport in the PG (Chanut-Delalande et al., 2014; Enya et al., 2014). The primary reaction catalyzed by GSTs is the conjugation of the reduced form of L-γ-glutamyl-L-cysteinylglycine or glutathione (GSH) to substrates. Consistent with the fact that Nobo is a GST, *gclc*, encoding an enzyme required for the *de novo* synthesis of GSH, was shown to partly phenocopy *nobo* mutants in *D. melanogaster* (Enya et al., 2017). Although the endogenous substrates of Nobo remain unclear, a high-throughput chemical screen and X-ray crystallography revealed that several small chemical compounds can be inserted into the putative ligand-binding pocket of Nobo, including the mammalian female sex hormone, 17β-estradiol (Fujikawa et al., 2015; Koiwai et al., 2020, 2021). This implies that a steroid could also be an endogenous substrate of Nobo, but future research is needed to clarify this point.

Ecdysteroids play a pivotal role in the development of arthropods. Consistent with this fact, ecdysteroid biosynthetic enzyme genes are mostly well conserved in the genomes of insects as well as other arthropods (Markov et al., 2009; Schumann et al., 2018). However, some enzymes are curiously missing from specific taxonomic groups of arthropods. For example, the genomes of Myriapoda and Chelicerata lack *phm* (Schumann et al., 2018) and thus the ability to perform carbon-25 hydroxylation of ecdysteroids. Consequently, it is thought that the main ecdysteroid is ponasterone A instead of 20E in these species. Likewise, no clear orthologs of *nvd* have been identified in any coleopteran species (R.N., unpublished observation). This is paradoxical, as the red flour beetle *Tribolium castaneum* requires sterols for larval development (Fraenkel and Blewett, 1943a,b). Orthologs of *nobo* are well conserved in dipteran and lepidopteran species, but are not found in other insect species (Enya et al., 2014; Koiwai et al., 2020). More strikingly, *spok* and *Cyp6t3* are found only in *Drosophilidae* species (Ono et al., 2006; Sztal et al., 2007; Ou et al., 2011). These facts have suggested that some ecdysteroid biosynthetic enzymes have evolved and are maintained in very limited clades, as reported in a recent comprehensive phylogenetic analysis (Schumann et al., 2018).

## Transcription Factors to Regulate the Expression of Halloween Genes

**FIGURE 2:**
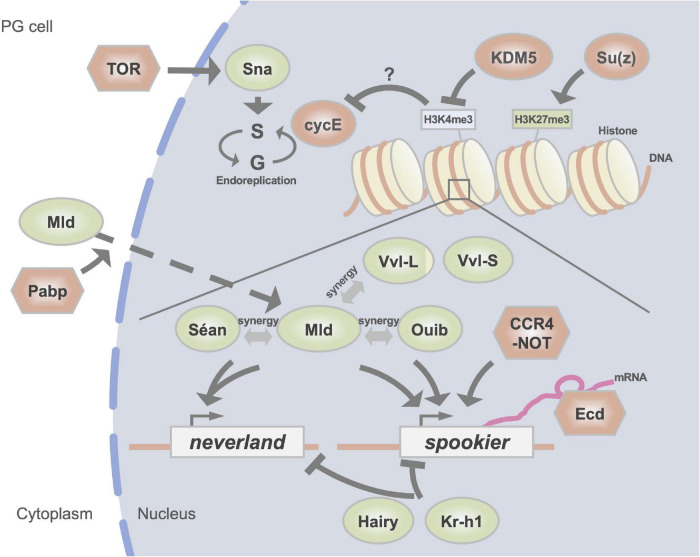
A schematic illustration of regulators of the expression of Halloween genes in the PG. DNA-binding transcription factors are indicated in green circles. Red circles represent histone modification enzymes and cell cycle regulators. Other factors are indicated in red hexagons. H3K4me3 and H3K27me3 indicate trimethylation of the fourth and 27th lysine residues of histone H3, respectively.

The temporal profiles of Halloween genes have been found to correlate well with the changes in the 20E titer during larval development (Niwa and Niwa, 2014b; Yamanaka and Okamoto, 2020). In addition, all known Halloween genes, except for *shd*, display high tissue specificity, as they are predominantly expressed in the PG and ovaries (Niwa and Niwa, 2014a; Kamiyama and Niwa, 2021b). Such temporally dynamic and spatially restricted expression profiles of the Halloween genes imply a tight transcriptional control network. To date, many transcription factors (TFs) have been reported to be responsible for the expression of Halloween genes (Niwa and Niwa, 2016b). In addition, posttranscriptional and epigenetic regulation are crucial for the expression of Halloween genes. All known validated and putative ecdysteroidogenic TFs required in PG are listed in Table 2. The modes of action of some ecdysteroidogenic TFs are illustrated in Figure 2.

**TABLE 2:** A list of ecdysteroidogenic transcription factors.

TF name	Protein family	Reported Halloween genes whose expression levels are affected	Positive (+) or negative (-) effects on targets	Organisms analyzed in published studies	References
Antp	Homeobox	*phm*	+	*Bombyx mori*	Meng et al., 2015
Br-C	C_2_H_2_ zinc finger	*phm, dib, sad*	+ (Br-Z4) – (Br-Z1)	*Drosophila melanogaster*	Xiang et al., 2010; Moeller et al., 2013
CncC	Basic leucine zipper	*nvd, spok, phm, dib, sad*	+	*D. melanogaster*	Deng and Kerppola, 2013
CTCF	C_2_H_2_ zinc finger	*nobo, spok, sad*	+	*D. melanogaster*	Fresán et al., 2015
DHR3	Nuclear receptor	*phm, dib, sad*	+	*D. melanogaster*	Parvy et al., 2014
DHR4	Nuclear receptor	*Cyp6t3*	–	*D. melanogaster*	Ou et al., 2011
Keap1	BTB	*nvd, spok, phm*	+	*D. melanogaster*	Deng and Kerppola, 2013
E75	Nuclear receptor	*phm*	+	*D. melanogaster*	Bialecki et al., 2002; Cáceres et al., 2011; Parvy et al., 2014
EcR	Nuclear receptor	*sro, phm, dib, sad*	+	*D. melanogaster*	Moeller et al., 2013; Parvy et al., 2014
FoxO	Fork head box	*phm, dib spo*	–	*D. melanogaster* *Tribolium castaneum*	Koyama et al., 2014; Lin et al., 2018
βFtz-f1	Nuclear receptor	*phm, dib, sad*	+	*D. melanogaster*	Parvy et al., 2005, 2014
hairy	Basic helix-loop-helix	*sro, spok, phm, dib, sad*	–	*D. melanogaster*	Yang et al., 2021
Kni	C_2_H_2_ zinc finger	*nvd, spok, sro, phm, dib, sad*	+	*D. melanogaster*	Danielsen et al., 2014
Kr-h1	C_2_H_2_ zinc finger	*nvd, sro, spok, Cyp6t3, phm, sad*	–	*D. melanogaster*	Zhang et al., 2018
Mld	C_2_H_2_ zinc finger	*nvd, spok, sro*	+	*D. melanogaster*	Ono et al., 2006; Danielsen et al., 2014; Uryu et al., 2018
Ouib	C_2_H_2_ zinc finger	*spok*	+	*D. melanogaster*	Komura-Kawa et al., 2015
Scr	Homeobox	*nvd*	+	*B. mori*	Daimon et al., 2020
Séan	C_2_H_2_ zinc finger	*nvd*	+	*D. melanogaster*	Uryu et al., 2018
Sna	C_2_H_2_ zinc finger	*nvd, sro, spok, phm, dib, sad*	+	*D. melanogaster*	Zeng et al., 2020
Usp	C_2_H_2_ zinc finger	*phm, dib*	–	*D. melanogaster*	Koyama et al., 2014
Vvl/POU-M2	POU-Hox	(Vvl-L&S) *nvd, spok, sro, phm, dib, sad* (Vvl-L) *nvd, spok, dib, sad spo, phm phm, dib, sad spo, dib*	+	*D. melanogaster* *D. melanogaster* *T. castaneum* *B. mori* *Oncopeltus fasciatus*	Cheng et al., 2014; Danielsen et al., 2014; Meng et al., 2015; Sarwar et al., 2020; Zhao et al., 2021

### Nuclear Receptors

The first identified TF that influences the levels of expression of validated Halloween genes is βFtz-f1, a critical regulator of insect metamorphosis in many tissues (Parvy et al., 2005). A study on *D. melanogaster* showed that the protein levels of Phm and Dib were significantly reduced following the loss of β*Ftz-f1* function in PG cells (Parvy et al., 2005). It was also revealed that the activity of βFtz-f1 in PG is modulated by SUMOylation (Talamillol et al., 2008; Talamillo et al., 2013). Notably, βFtz-f1 is a nuclear receptor (NR) homologous to Ad4BP/SF-1, a key TF for steroidogenic genes in mammalian cells (Morohashi et al., 2013). Moreover, mammalian Ad4BP/SF-1 is also SUMOylated, resulting in the modulation of the expression of steroidogenic genes, thus influencing adrenocortical steroidogenesis (Suda et al., 2011). These data imply that the role of βFtz-f1/Ad4BP/SF-1 in steroidogenesis and the posttranslational modification of them are evolutionarily conserved between insects and vertebrates, at least in part.

β*Ftz-f1* is well known to be transcriptionally induced by a surge of 20E during development (Yamada et al., 2000). Given the fact that 20E signaling plays a pivotal role in the sequential induction of not only β*Ftz-f1* but also other NR genes (Thummel, 2002), it is plausible that other NRs are also involved in the transcriptional regulation of Halloween genes. Indeed, this is the case, as 20E-responsive NR genes other than βFtz-f1 also regulate the expression of Halloween genes in the PG.

One such 20E-responsive NR is Ecdysone receptor (EcR) itself (Moeller et al., 2013; Parvy et al., 2014). Dynamic feedback regulation of 20E-EcR signaling in the PG is important for ecdysteroid biosynthesis. During the larval stages, reduced 20E-EcR signaling in the PG decreases the expression of Halloween genes, leading to a delay in the larva-to-prepupa transition (Moeller et al., 2013). This observation indicates that 20E has a positive feedback effect on PG, rapidly amplifying its own synthesis to trigger pupariation. In contrast, after pupariation, reduced 20E-EcR signaling increases the expression of Halloween genes, leading to metamorphosis. These findings indicate that 20E conversely has a negative feedback signal that ensures a decline in the level of 20E in the prepupa-to-pupa transition. These opposite effects are partly regulated by the 20E-responsive Broad (*Br*) gene, as described in the next section.

Downstream of 20E-EcR signaling, other 20E-responsive NRs, E75, βFtz-f1, and DHR3, regulate ecdysteroid biosynthesis in the PG (Bialecki et al., 2002; Cáceres et al., 2011; Moeller et al., 2013; Parvy et al., 2014). E75 and βFtz-f1 enhance ecdysteroid biosynthesis, whereas DHR3 suppresses it. In addition, loss of function of these TFs influences the expression of a part of Halloween genes (Table 1). Moreover, the expression of *E75* is affected by *EcR*, whereas the expression of *DHR3* is affected by E75 and βFtz-f1. These findings suggest that the NR-mediated 20E-responsive gene expression cascade is involved in the control of the expression of Halloween genes.

DHR4, which is known as a 20E-responsive gene (King-Jones et al., 2005), negatively regulates ecdysteroid biosynthesis in the PG (Ou et al., 2011). PG-specific DHR4 knockdown in the larval stage caused a higher ecdysteroid titer and precocious puparium formation. Interestingly, the subcellular localization of DHR4 is temporally regulated by prothoracicotropic hormone (PTTH), the important and most classical humoral factor that stimulate the biosynthesis and secretion of ecdysteroids in the PG (Kawakami et al., 1990; McBrayer et al., 2007; Rewitz et al., 2009b; Ou et al., 2011). When PTTH signaling is active in the PG, DHR4 is exported from the nucleus to the cytoplasm, leading to the suppression of DHR4-mediated gene expression. One of the essential targets of DHR4 is *Cyp6t3*.

Another 20E-responsive NR, Ultraspiracle (Usp), which is a heterodimer partner of EcR, is also involved in the expression of Halloween genes (Koyama et al., 2014). This point is described in more detail later.

### Broad-Complex

The Broad-complex (Br-C) gene belongs to the Br-C, Tramtrack, Bric-a-brac/Poxvirus, and zinc finger (BTB/POZ) family of C_2_H_2_-zinc finger TFs. It is well known that *Br-C* is crucial for the appropriate regulation of many 20E-inducible genes as larvae enter metamorphosis (Belles, 2020). In *D. melanogaster*, *Br-C* expresses 4 different isoforms, designated *Br-Z1* to *Br-Z4*. All *Br-C* isoforms are expressed in the PG (Zhou et al., 2004). In addition, overexpression of any of the isoforms in the PG prevents larval molting or pupariation to varying degrees, which is consecutively rescued by administration of 20E (Zhou et al., 2004). These results suggest an inhibitory effect of Br-C on ecdysteroid biosynthesis in the PG. However, in reality, this situation is not very simple.

PG-specific RNAi experiments revealed that, among these isoforms, Br-Z1 and Br-Z4, not Br-Z2 or Br-Z3, are involved in the regulation of ecdysteroid biosynthesis in the PG (Xiang et al., 2010; Moeller et al., 2013). Interestingly, Br-Z4 regulates the expression of Halloween genes positively, whereas Br-Z1 negatively. The functional differences between Br-Z4 and Br-Z1 are also reflected by the different responses to 20E, as the expression of *Br-Z4* is more acutely induced by 20E than *Br-Z1* (Moeller et al., 2013). In fact, the 20E-EcR-mediated positive and negative feedback regulation of ecdysteroid biosynthesis in the PG, was demonstrated to be dependent on the differential functions of Br-Z4 and Br-Z1. The positive feedback regulatory mechanism requires Br-Z4 to induce the expression of Halloween genes in the late third larval instar, whereas the negative feedback regulatory mechanism requires Br-Z1 to suppress the expression of Halloween genes in the prepupal and pupal stages (Moeller et al., 2013). Taken together, Br-C plays a crucial role in the EcR-dependent switching from positive to negative feedback regulation, shaping the pulses of the 20E titer during the larval-to-pupal transition.

### Molting Defective, Séance, and Ouija Board

Besides Br-C, other C_2_H_2_-zinc finger TFs, namely Molting defective (Mld), Séance (Séan), and Ouija board (Ouib) are also involved in ecdysteroid biosynthesis in the PG. All 3 TFs belong to the zinc finger associated domain (ZAD)-C_2_H_2_ zinc finger family.

Mld was identified from a mutant with a larval molting defect and low 20E titer (Neubueser et al., 2005). In addition, a classical temperature-sensitive mutant *lethal(3)dts3*, which shows ecdysteroid deficiency at the restrictive temperature (Walker et al., 1987), has been recognized as an allele of *mld* (Simon et al., 2003; Ishimoto et al., 2013). Studies have also shown that loss of *mld* function in the PG specifically impairs the expression of *nvd* and *spok*, but not that of other Halloween genes (Neubueser et al., 2005; Ono et al., 2006; Danielsen et al., 2014). Importantly, the lethal phenotype of loss-of-*mld* function mutants is rescued by the simultaneous expression of both *nvd* and *spo* (the paralog of *spok*) (Uryu et al., 2018). These data indicate that, at least during larval development, the essential downstream targets of Mld are only *nvd* and *spok*.

Two other TFs, Séan (also known as Numerous disordered muscles; Dobi et al., 2014) and Ouib, are also essential for the transcriptional regulation of *nvd* and *spok* (Komura-Kawa et al., 2015; Niwa and Niwa, 2016a; Uryu et al., 2018). Strikingly, loss-of-function mutations in *séan* or *ouib* result in a larval-arrest phenotype with a dramatic reduction in the expression of *nvd* and *spok*, respectively, but not in that of other Halloween genes. More importantly, this lethal phenotype is rescued by expression of *nvd* or *spo* alone, respectively (Komura-Kawa et al., 2015; Uryu et al., 2018). These data indicate that, at least during larval development, only *nvd* and *spok* are essential downstream targets of Séan and Ouib, respectively. It should also be noted that among all identified ecdysteroidogenic TFs, Ouib is the only ecdysteroidogenic TF that is specifically localized in the PG and not in other tissues (Komura-Kawa et al., 2015).

Further analyses using a cell culture system have revealed that these 3 TFs cooperatively control the expression of *nvd* and *spok*. Coexistence of both Mld and Séan in cultured cells results in a drastic induction of *nvd* promoter-driven reporter gene expression compared with that of cells treated with Mld or Séan alone (Uryu et al., 2018). Similarly, coexistence of both Mld and Ouib dramatically induces *spok* promoter-driven reporter gene expression compared with Mld or Ouib alone (Komura-Kawa et al., 2015). These studies have revealed the unique feature of the cooperative role of Mld, Séan, and Ouib in regulating a very small subset of genes, that is, *nvd* and *spok*. As the ZAD domain is known to be protein-protein interaction domain (Jauch et al., 2003), this suggest that a physical interaction between Mld and Séan or between Mld and Ouib is possible. However, such protein-protein interactions have not been reported to date. Therefore, it remains unclear how this cooperativity is achieved (Uryu et al., 2018).

Of note, the gene loci of *nvd* and *spok*, but not that of other Halloween genes, are located in the pericentric constitutive heterochromatin region in the *D. melanogaster* genome (Ono et al., 2006; Yoshiyama et al., 2006; Fitzpatrick et al., 2011). Although constitutive heterochromatin has long been thought to be transcriptionally inert in cells, a number of studies have revealed that it contains active genes essential for viability (Dimitri et al., 2009). To date, Mld, Séan, and Ouib represent the first example of a set of TFs that regulate genes located in constitutive heterochromatin in a spatiotemporal-specific manner.

From an evolutionary point of view, *mld*, *séan*, and *ouib* are also unique because their orthologs are found only in genomes of *Drosophilidae* species (Neubueser et al., 2005; Ono et al., 2006; Komura-Kawa et al., 2015; Uryu et al., 2018). This trend is partly consistent with the fact that *spok* (but not *nvd*) is only conserved in genomes of *Drosophilidae* species (Ono et al., 2006). Therefore, it has been suggested that some essential ecdysteroidogenic TFs might have rapidly evolved only in very limited insect clade(s).

### Ventral Vein Lacking

Ventral vein lacking (Vvl), also known as Ventral veinless or Drifter (Drf), belongs to the POU homeobox TF family and plays versatile roles in cell fate specification, organ patterning, and immune defense. As a TF that regulates the expression of Halloween genes, Vvl was independently identified in studies using *D. melanogaster* (Danielsen et al., 2014) and the red flour beetle *Tribolium castaneum* (Cheng et al., 2014). The role of *vvl* orthologs in ecdysteroid biosynthesis has also been confirmed in other insects, such as the silkworm *Bombyx mori* (Meng et al., 2015) and the milkweed bug *Oncopeltus fasciatus* (Sarwar et al., 2020). In *D. melanogaster*, loss of *vvl* function results in the reduced expression of most Halloween genes (Danielsen et al., 2014). Conversely, overexpression of the *B. mori* ortholog of *vvl*, known as POU-M2, can induce the expression of some Halloween genes in *B. mori* culture cells (Meng et al., 2015). In addition, POU-M2 was demonstrated to directly bind to potential *cis-*regulatory elements in the *phm* promoter region. These data suggest that the function of Vvl in regulating the expression of Halloween genes is conserved in insects.

A recent study demonstrated that the *vvl* gene produces long and short isoforms through a stop codon readthrough process in the PG during larval development (Zhao et al., 2021). Stop codon readthrough is a phenomenon in which near-cognate tRNAs are able to decode stop codons as sense codons, resulting in a read-frame extension even in a conventional 3′ untranslated region. While the error rate of translational termination should be less than 0.1%, surprisingly, the stop codon readthrough of *vvl* mRNA reaches a rate of 50% in the PG (Zhao et al., 2021). Consequently, the C-terminal extension of Vvl produces a long isoform, which possesses an extra 286 amino acid residues compared with the short isoform (427 amino acid length) encoded by the conventional *vvl* coding sequence. Loss-of-function mutations that lead to the specific lack of the long isoform have been associated with pupariation delay and decreased expression of *nvd*, *spok, dib*, and *sad*, even in the presence of the short isoform.

A chromosomal deletion of a human homolog of *vvl*, known as POU3F2, has been associated with hypogonadotropic hypogonadism and adrenal insufficiency (Bonaglia et al., 2008; Izumi et al., 2013). Therefore, it is suggested that the Vvl/POU3F2 family participates in the conserved regulatory mechanisms of steroidogenesis between insects and vertebrates.

### CncC and Keap1

Cap-n-collar (CncC), the *D. melanogaster* ortholog of mammalian nuclear factor erythroid 2-related factor 2 (Nrf2), and its cofactor Kelch-like ECH associated protein 1 (Keap1) are evolutionary conserved key TFs that regulate xenobiotic responses and redox homeostasis (Ulasov et al., 2021). Although CncC-Keap1 signaling is indispensable for these biological processes, it has also been found to be involved in ecdysteroid biosynthesis (Deng and Kerppola, 2013). Studies have shown that PG-specific knockdown of *CncC* or *Keap1* leads to decreased expression of a wide variety of Halloween genes in the PG. In addition, accelerated pupariation induced by the overexpression of a constitutively activated oncogenic *RAS* is suppressed by knocking down *CncC*. Considering that RAS is necessary for PTTH signaling (Rewitz et al., 2009b; Pan et al., 2020; Kannangara et al., 2021), CncC seems to be downstream of the PTTH-RAS pathway. In addition, a more recent study has also found that loss of function of *Keap1* results in the reduction in the expression of several Halloween genes in the ovary, another ecdysteroid biosynthetic organ (Carlson et al., 2022).

### FoxO-Ultraspiracle

Forkhead box transcription factor class O (FoxO) regulates the metabolism, cellular proliferation, stress tolerance, and lifespan in most animals, including insects (Calnan and Brunet, 2008). *In D. melanogaster* PG, FoxO functions as a negative regulator of the expression of Halloween genes just before the early generation of a small peak of 20E in the third (final) larval instar of *D. melanogaster* (Koyama et al., 2014). This small peak of 20E has been proposed to induce a developmental transition known as the critical weight (Mirth and Shingleton, 2012). Critical weight is the minimum body weight of larvae that is essential for the transition to pupa. Before critical weight, FoxO is localized in the nuclei of PG, suppressing the expression of Halloween genes, and thus of ecdysteroid biosynthesis. In contrast, after critical weight, FoxO is exported to the cytoplasm and excluded from the nuclei, resulting in the generation of a small 20E peak. Interestingly, FoxO has been found to be localized in the nuclei of larvae that are reared under starved conditions at the same time point. As they cannot generate this small 20E peak, they consequently cannot grow beyond the critical weight (Koyama et al., 2014). These data suggest that nuclear localization of FoxO determines the critical weight state attainment by suppressing the expression of the Halloween genes before this time point. Moreover, FoxO is known to physically interact with the nuclear hormone receptor Usp, which is also required for the suppression of the expression of Halloween genes (Koyama et al., 2014).

Besides *D. melanogaster*, the role of FoxO in ecdysteroid biosynthesis in the PG was also demonstrated in *T. castaneum*. RNAi of *FoxO* results in a delay of ecdysteroid biosynthesis (Lin et al., 2018). In the RNAi animals, expression of *spo* is significantly delayed, whereas that of *phm*, *dib*, *sad*, or *shd* is not.

It is well known that the nuclear localization of FoxO is negatively regulated by the activity of insulin/insulin-like growth factor signaling (IIS) (Calnan and Brunet, 2008). Moreover, IIS has been demonstrated to enhance ecdysteroid biosynthesis in *D. melanogaster* PG (Colombani et al., 2005). Therefore, FoxO, and possibly Usp, might be crucial for the suppression of the expression of Halloween genes before the critical weight when the activity of IIS is low (Koyama et al., 2014). As the critical weight 20E peak responds to environmental cues, including nutritional status (Mirth and Shingleton, 2012), both IIS and FoxO-Usp seem to play an essential role in the developmental transition influenced by nutritional availability.

### Homeotic/Hox Transcription Factors

Homeotic (Hox) transcription factors confer segmental identity along the anteroposterior axis of insect embryos (Mallo and Alonso, 2013). Sex comb reduced (Scr), a homeotic transcription factor, is required for the differentiation and morphogenesis of PG (Sánchez-Higueras et al., 2014). Of note, PG has a homologous origin with the respiratory tracheal system in *D. melanogaster* and is derived from the primordium in the labial segment of the embryo. Scr cooperates with signal transducer and activator of transcription (STAT), inducing the expression of *vvl* in the labial patch. Subsequently, a subgroup of *vvl*-expressing cells activates the Zn-finger gene Snail (Sna), a key regulator of the epithelial-mesenchymal transition. The differentiated PG cells eventually express the specific marker TF gene *spalt*. These TF associations appear to be essential for establishing the cellular status of PG cells (Sánchez-Higueras et al., 2014).

In addition to shaping the segment-specific establishment of the PG, some homeotic transcription factors also regulate the expression of Halloween genes in differentiated PG. In particular, 2 homeotic transcription factors, Antennapedia (Antp) and Scr, regulate the expression of Halloween genes in *B. mori* (Meng et al., 2015; Daimon et al., 2020). *Antp* is highly expressed in the *B. mori* PG, and its temporal expression pattern in the PG is positively correlated with that of Halloween genes. In addition, Antp directly binds to potential *cis-*regulatory elements in the *phm* promoter region (Meng et al., 2015).

*Scr* was identified as a gene responsible for *Moltinism B. mori* strains (Daimon et al., 2020). *Moltinism* strains molt 3, 4, or 5 times, which is determined by allelic variations at a single autosomal locus. Positional cloning has revealed that this locus overlaps with the *Scr* gene region. While *Moltinism* strains do not exhibit any abnormalities in segmental identity, mutations in these strains induce abnormal expression of *Scr* in the PG. Inhibiting the function of Scr in the PG upregulates the expression of *nvd*, hence increasing the basal ecdysteroid titer during the early fifth larval instar. This result suggest that increased basal ecdysteroid titer leads to the extra molt to the sixth instar larvae, but not pupae (Daimon et al., 2020). This interpretation resembles the “leaky prothoracic glands” hypothesis, which proposes that the effects of low levels of ecdysone over a long period trigger the molt, similar to high levels of ecdysone over a brief period (Nijhout, 1976; Callier and Nijhout, 2011). Taken together, these results suggest that Scr plays a role in controlling the number of proper molts through the suppression of the expression of *nvd* in the PG.

### Period and Timeless

Period (Per) and Timeless (Tim) are indispensable TFs that consist of the central components of the circadian pacemaker clock gene regulatory network in insects (Dubowy and Sehgal, 2017). Both Per and Tim oscillate in the PG of larvae (Di Cara and King-Jones, 2016) and pupae (Myers et al., 2003; Morioka et al., 2012). PG-specific knockdown of either *per*, *tim*, or other clock component genes results in the larval-arrest phenotype (Di Cara and King-Jones, 2016). In addition, PG-specific overexpression of *tim* affects eclosion timing (Myers et al., 2003). Moreover, at least in the larval stage, both Per and Tim are required for the transcriptional upregulation of Halloween genes (Di Cara and King-Jones, 2016). These data suggest that Per and Tim are part of the PG circadian clock that influences developmental progress via the transcriptional regulation of Halloween genes.

In some insects, molting is determined by circadian “gating,” where a ecdysteroid pulse is delayed by 24 h if the appropriate time window is missed (Truman and Riddiford, 1974; Mizoguchi and Ishizaki, 1982). However, not only molting in *D. melanogaster* is not dependent on such gating, but also its genetic null mutants of *per* and *tim* are viable (Zerr et al., 1994; Myers et al., 1995). Nevertheless, paradoxically, PG-specific knockdown of either *per* or *tim* results in a larval-arrest phenotype. This discrepancy might be explained by the hypothesis that PG-specific knockdown against circadian components causes dis-synchronization between the PG and external timing signals, leading to catastrophic effects on ecdysteroid biosynthesis (Di Cara and King-Jones, 2016). Indeed, Tim is known to couple the circadian machinery directly to IIS and PTTH signaling in the PG. Moreover, the active IIS directly modulates the function of Tim, suggesting that the local clock in the PG is normally synchronized with systemic insulin cues (Di Cara and King-Jones, 2016). Because both PTTH and systemic IIS are themselves under circadian control, the desynchronization of the local endocrine clock in the PG with external circadian cues appears to be the primary cause for the failure in ecdysteroid biosynthesis.

In some insects, including the eri silk moth *Samia cynthia ricini*, the PG is thought to contain a photoreceptor that regulates timed developmental events (Mizoguchi and Ishizaki, 1982). A recent RNA-seq study has revealed that *cryptochrome* (*cry*), which encodes the circadian transcriptional repressor essential for photoentrainment, is expressed in *B. mori* PG (Bian et al., 2019). Moreover, the oscillation in the expression of *tim* in the PG has been shown to be diminished or weakened in the *cry* mutant background in *D. melanogaster* (Myers et al., 2003; Morioka et al., 2012). However, PG-specific knockdown of *cry* is not associated with any developmental timing/arrest phenotype in *D. melanogaster* (Di Cara and King-Jones, 2016). Therefore, the role of *cry* in the PG circadian clock remains unclear.

### Snail

Sna is another TF exhibiting versatile functions during insect development (Nieto, 2002). As described in the section “Homeotic/Hox transcription factors,” Sna is necessary for epithelial-mesenchymal transition in the PG. Moreover, Sna also plays an essential role in endoreplication, which is the replication of genomic DNA that is not followed by cell division, in the PG (Zeng et al., 2020). Sna shows a temporally fluctuating expression pattern in the PG of the third instar in *D. melanogaster* larvae. Interestingly, knocking down *sna* leads to PG cells failing to undergo the endoreplication cycle, thus resulting in a significant reduction in their nuclear size. In addition, *sna* knockdown significantly suppress the expression of almost all Halloween genes (Zeng et al., 2020).

Of note, PG cells grow continuously by endoreplication throughout the larval stage. Inhibition of endoreplication, specifically in the PG, causes developmental arrest in the larval stage (Ohhara et al., 2017). Endoreplication in PG cells is essential for the progression of the third instar development in *D. melanogaster* after critical weight. Blocking endoreplication in the PG causes a significant reduction in the 20E titer because of the suppression in the expression of most Halloween genes. Moreover, progression of endoreplication in PG cells requires the upregulation in the expression of *cyclin E* by the nutrient sensor target of rapamycin (TOR) signaling. Therefore, nutrient-dependent endoreplication is essential for critical weight attainment, resulting in the strong expression of Halloween genes timely before pupariation for the initiation of metamorphosis (Ohhara et al., 2017, 2019). Conclusively, Sna is considered to regulate the expression of Halloween genes by coordinating endoreplication in a TOR-dependent manner in the PG; however, the essential downstream targets of Sna remain unclear (Zeng et al., 2020).

### Hairy and Krüppel-Homolog 1

A basic helix-loop-helix (bHLH) transcription factor, Hairy, is known as one of the signal transducers of the sesquiterpenoid insect hormone, juvenile hormone (JH). A recent study has revealed that Hairy acts as a negative regulator of the expression of Halloween genes in a JH-dependent manner in *D*. *melanogaster* and *B. mori* (Yang et al., 2021). Curiously, the expression of *hairy* is suppressed by trimethylation of histone H3 on lysine 27 (H3K27me3), which is catalyzed by Polycomb repressive complex 2 (PRC2) with histone methyltransferase activity. H3K27me3 level dynamically increases during the final larval instar of *D. melanogaster* and *B. mori*, at which 20E titer is elevated. Consistent with the correlation between H3K27me3 level and 20E titer, PG-specific knockdown of *suppressor of zeste 12* (*Su(z)12*), encoding a component of PRC2, results in reduction in H3K27me3, diminishing ecdysteroid biosynthesis and disturbs the larval–pupal transition in *D. melanogaster* (Yang et al., 2021). Mechanistically, H3K27me3 targets *hairy* to repress its transcription in the PG, leading to an upregulation in the expression of Halloween genes. Moreover, the application of a JH mimic to PG decreases both the levels of H3K27me3 and the expression of *Su(z)12*. An attenuation in the expression of some Halloween genes has also been observed in *B. mori* injected with the chemical inhibitor of PRC2 (Yang et al., 2021). Taken together, these data suggest that PRC2-mediated H3K27me3 at the *hairy* locus in the PG is negatively regulated by JH and required for ecdysteroid biosynthesis during the larval–pupal transition.

The suppressive function of JH on ecdysteroid biosynthesis in the PG is reminiscent of the downregulation of Halloween genes by another JH signal transducer, Krüppel-homolog 1 (Kr-h1) (Zhang et al., 2018). These studies provide valuable insights into JH-mediated ecdysteroid biosynthesis.

### Other Transcription Factors and Epigenetic Regulators

A number of other TFs are also involved in ecdysteroid biosynthesis in the PG.

Knirps (Kni) is a well-known gap gene product essential for the segmentation process during early embryogenesis (Jaeger, 2011). Kni was identified through *in silico* analyses of *cis*-regulatory elements as a candidate TF that can bind to the enhancer regions of *spok*, *phm*, and *dib* (Danielsen et al., 2014). *kni* is strongly expressed in the larval PG cells, and PG-specific knockdown of *kni* results in a larval-arrest phenotype.

CCCTC-binding factor (CTCF), a highly conserved insulator protein, has also been reported to regulate ecdysteroid biosynthesis (Fresán et al., 2015). PG-specific knockdown of *CTCF* causes low transcriptional levels of *nobo*, *spok*, and *sad*. Curiously, puparium formation delay caused by PG-specific *CTCF* knockdown is rescued by administration of both cholesterol and 20E, but not by cholesterol or 20E alone. Therefore, CTCF might play a role not only in ecdysteroid biosynthesis, but also in cholesterol homeostasis in the PG.

Without children (woc) is a C_2_H_2_-type zinc finger protein. Homozygous mutations of *woc* results in larval lethality with ecdysteroid deficiency (Wismar et al., 2000). Because administration of 7-dehydrocholesterol partially rescues this mutant (Warren et al., 2001), it has been hypothesized that Woc activates the transcription of a gene involved in 7,8-dehydrogenation of cholesterol to produce 7dC. However, it is unlikely that Woc regulates the expression of *nvd*, encoding the cholesterol 7,8-dehydrogenase, in PG (Yoshiyama et al., 2006). The actual downstream target of Woc is currently unknown.

HLH54F is a basic helix-loop-helix TF gene that is predominantly expressed in the PG of both *D. melanogaster* and *B. mori* (Namiki et al., 2009). However, no validated targets of HLH54F in PG have been reported.

The chromatin remodeling factor lysine demethylase 5 (KDM5) is also known to be involved in ecdysteroid biosynthesis (Drelon et al., 2019). KDM5 is a trimethylated histone H3 on lysine 4 (H3K4me3) demethylase. A loss–of-function *KDM5* genetic mutant exhibits severe pupariation delay (Drelon et al., 2019). Consistent with this phenotype, the 20E titer and the level of expression of Halloween genes are decreased in this mutant. Unexpectedly, although KDM5 is believed to participate in the H3K4me3 demethylation process in almost all cells, the PG-specific forced expression of *KDM5* rescued the pupariation delay and decreased ecdysteroid titer in the *KDM5* genetic mutant. In addition, the pupariation delay of *KDM5* genetic mutants is also partially rescued by the PG-specific forced expression of the PTTH receptor gene *torso* or *cycE*. These data indicate that the KDM5-mediated demethylation of H3K4me3 is required for proper gene expression in PG cells, at least sustaining PTTH signaling and endoreplication (Drelon et al., 2019).

Recent RNA-seq analyses have reported the predominant expression of other TFs in the ring gland, an endocrine tissue complex containing PG in higher dipteran species; however, their functions in the PG have not been elucidated (Ou et al., 2016; Christesen et al., 2017; Nakaoka et al., 2017; Moulos et al., 2018; Bian et al., 2019). These uncharacterized factors include CG6163 (MADF-BESS domain), CG33557 (bHLH), Escargot (C_2_H_2_ zinc finger), Hand (bHLH), Mesoderm-expressed 2 (MADF-BESS domain), Pou domain motif 3 (Pdm3) (POU-Hox), and Silk gland factor 3 (POU-Hox). It would be intriguing to examine whether any of these TFs are produced in the PG and influence the expression of Halloween genes.

## Posttranscriptional Regulators of the Expression of Halloween Genes

Some studies have revealed that posttranscriptional regulation, such as splicing and polyadenylated [poly(A)]-tail modification, are also important in modulating the expression of Halloween genes.

The role of splicing in the expression of Halloween genes has mostly been elucidated in a study on *spok* and *ecdysoneless* (*ecd*) in *D. melanogaster*. *Ecd* was originally discovered as a genetic mutant resulting in ecdysteroid deficiency (Garen et al., 1977; Gaziova et al., 2004). It encodes an evolutionarily conserved protein that acts as a member of the spliceosome complex. As Ecd seems to regulate only a part of the splicing process, its dysfunction was reported to impact the splicing of *spok* but not that of *phm* pre-mRNA among Halloween genes in the PG (Claudius et al., 2014). Notably, the larval-arrest phenotype of *ecd* loss-of-function mutants is rescued by overexpression of the human ortholog of *ecd* (Claudius et al., 2014), suggesting that the function of Ecd as a regulator of splicing is conserved in humans as well.

Poly(A)-tail modifiers are also involved in the regulation of the expression of *spok.* Loss-of-function of a key subunit of the CCR4-NOT complex Pop2 and poly(A)-binding protein (Pabp) decreases in the expression of *spok* (Zeng et al., 2018; Kamiyama et al., 2020). CCR4-NOT is an mRNA deadenylation complex. Generally, it is thought that a longer poly(A)-tail contributes to mRNA stability. However, loss of the function of *Pop2* in larvae leads to a larval-arrested phenotype, extended poly(A)-tail of *spok* mRNA, and decreased levels of *spok* mRNA (Zeng et al., 2018). Therefore, the CCR4-NOT complex might regulate the transcription of *spok* directly in the PG. Loss-of-function of *Pabp* has also been reported to lead to a decrease in the expression of *spok* (Kamiyama et al., 2020). In PG-specific *pabp*-knockdown animals, Mld, which is indispensable for the expression of *spok*, is abnormally accumulated in the cytoplasm of the PG. Such abnormal accumulation is not observed in the case of Vvl and Pdm3, which are also expressed in the PG. These data imply that Pabp contributes specifically to the nuclear localization of Mld, while it is unclear whether it regulates the subcellular localization of Mld directly or indirectly. Of note, PG-specific knockdown of the nuclear poly(A) polymerase gene *hiiragi* also causes developmental arrest in the larval stage (Danielsen et al., 2016). Taken together, these data suggest that poly(A)-related proteins are specifically involved in the regulation of the expression of Halloween genes.

## Future Perspectives and Remaining Questions

In the last 16 years, since βFtz-F1 was identified as an ecdysteroidogenic TF in the PG in 2005 (Parvy et al., 2005), many ecdysteroidogenic TFs (Table 2) have been identified and characterized, particularly in *D. melanogaster*. However, there are still many important issues to be solved for a better understanding of the transcriptional regulation of ecdysteroid biosynthesis. Below, we list some remaining questions that must be addressed in the future.

### How Multiple Ecdysteroidogenic Transcription Factors Coordinately Regulate the Expression of Halloween Genes?

To date, 20 types of TFs (Table 2) have been identified and characterized as ecdysteroidogenic TFs. However, it remains largely unclear whether and how these TFs interact or coordinate with each other to regulate the expression of Halloween genes. For example, in *D. melanogaster*, the expression of *phm* in the PG is transcriptionally influenced by at least 14 TFs, including Br-C, CncC, DHR3, Keap1, E75, EcR, FoxO, βFtz-F1, Hairy, Kni, Kr-h1, Sna, Usp, and Vvl (Table 2). The involvement of multiple TFs is also the case regarding other Halloween genes (Table 2). Currently, we do not understand why multiple TFs are required for the expression of a single Halloween gene. Does each ecdysteroidogenic TF differentially regulate Halloween genes in a context-dependent manner, such as nutritional availability? Does each ecdysteroidogenic TF cooperate with other TFs to induce the expression of Halloween genes? If so, do any of these ecdysteroidogenic TFs physically interact with each other? These questions must be answered for a comprehensive understanding of the role of TFs in the transcriptional regulation of Halloween genes. Notably, a recent study successfully conducted immunoprecipitation followed by mass spectrometry to identify physically bound proteins to a protein of interest from the PG samples (Huynh et al., 2019). This technology might be applicable for revealing the TF-mediated network to regulate Halloween genes.

It is also intriguing to examine which TF or combination of TFs is sufficient for the induction of the expression of Halloween genes. In mammals, Ad4BP/SF-1 is both necessary and sufficient for the induction and maintenance of the expression of steroidogenic genes. For example, overexpression of Ad4BP/SF-1 induces the differentiation of cultured stem cells into steroidogenic cells (Miyamoto et al., 2011). Moreover, transgenic expression of Ad4BP/SF-1 in mice leads to ectopic adrenal formation (Zubair et al., 2009). Among ecdysteroidogenic TFs, simultaneous forced expression of *mld* and *ouib* induces the expression of *spok* in *D. melanogaster* S2 cultured cells (Uryu et al., 2018). However, no studies have examined whether any other ecdysteroidogenic TFs, including βFtz-F1, are sufficient for inducing the expression of Halloween genes in non-steroidogenic cells in insects.

### How Do Identified Transcription Factors Contribute to Gene Expression Changes in Response to Extracellular Stimuli?

Ecdysteroid biosynthesis in the PG is tightly regulated by endocrine and autocrine humoral factors. Endocrine factors include PTTH (Kawakami et al., 1990; McBrayer et al., 2007), *Drosophila* insulin-like peptides (Dilps) (Colombani et al., 2005), serotonin (Shimada-Niwa and Niwa, 2014), Hedgehog (Rodenfels et al., 2014), Decapentaplegic (Setiawan et al., 2018), PDGF- and VEGF-related factors 2 and 3, and Jelly belly (Pan and O’Connor, 2021). Autocrine factors include octopamine (Ohhara et al., 2015) and epidermal growth factor (Cruz et al., 2020). While receptors for activin (Gibbens et al., 2011) and neuropeptide F (Kannangara et al., 2020) are also involved in ecdysteroid biosynthesis and the expression of Halloween genes in the PG, the source of these ligands is unclear. Most of these humoral factors affect the levels of expression of Halloween genes (Niwa and Niwa, 2016b; Pan et al., 2020; Texada et al., 2020; Kannangara et al., 2021). Therefore, the important issue in drawing a signaling network for controlling ecdysteroid biosynthesis is to understand which ecdysteroidogenic TFs act downstream of the extracellular stimuli-triggered signaling pathway. However, the mechanism by which these receptors regulate the transcription of Halloween genes remains largely unclear.

To date, DHR4 is the only good example of an ecdysteroidogenic TF acting downstream of PTTH signaling (Ou et al., 2011). A crucial function of DHR4 is to negatively regulate the expression of *Cyp6t3* when PTTH signaling is suppressed in the PG. However, DHR4 does not seem to be extensively involved in regulating the expression of any other Halloween gene (Ou et al., 2011), indicating that PTTH signaling must target other ecdysteroidogenic TFs. The PTTH receptor Torso is known to activate the Ras-ERK signaling pathway (Rewitz et al., 2009b). Therefore, it is likely that ERK might phosphorylate some ecdysteroidogenic TFs in the PG to regulate their transcriptional activity; however, a proteomic approach for the identification of phosphorylated proteins in the PG failed to detect the phosphorylation of identified ecdysteroidogenic TFs (Rewitz et al., 2009a). Alternatively, the Ras-ERK signaling pathway might affect epigenetic regulation in the PG, as ERK signaling is known to regulate gene expression by activating histone modification enzymes in mammals (Vicent et al., 2010).

### How Does Ploidy in the Prothoracic Gland Affect the Expression of Halloween Genes in the Prothoracic Gland?

While Sna is an indispensable TF that regulates endoreplication and the expression of Halloween genes (Zeng et al., 2020), it remains unclear the mechanism by which Sna is activated at a proper time point to evoke endoreplication in the PG. It has been suggested that the Warts-Yorkie signaling, IIS, and Ras-ERK signaling are regulators of endoreplication in *D. melanogaster* (Costa et al., 2021). These signaling pathways are active and essential for ecdysteroid biosynthesis in the PG (Colombani et al., 2005; Rewitz et al., 2009b; Moeller et al., 2017). Although TOR-dependent endoreplication in the PG requires Sna (Zeng et al., 2020), the roles of Warts-Yorkie and Ras-ERK have not been examined. Interestingly, an interaction has been suggested between chromatin remodeling by KDM5 and endoreplication (Drelon et al., 2019). Potentially, the progress of endoreplication in the PG might depend on the developmental timing-specific chromatin status during larval development.

The means by which the expression of Halloween genes is induced in PG cells with high ploidy is also unclear. A recent study has reported the use of a genetic screen for the identification of essential genes involved in ecdysteroid biosynthesis, but not the endoreplication process in the PG (Ohhara et al., 2019). The TFs and epigenetic regulators identified in this screen might help to understand the molecular mechanism of the expression of Halloween genes downstream of the endoreplication event.

### How Is Chromatin Modification in the Prothoracic Gland Changed During Development?

Chromatin status might be temporally changed during larval development in the PG, as the levels of H3K27me3 are gradually increased during the third larval instar (Yang et al., 2021). In fact, a large-scale RNAi screen was performed to knock down genes specifically in the PG and identified only the *Su(z)12* gene among 31 genes involved in methylation, demethylation, acetylation, deacetylation, and phosphorylation of histones (Danielsen et al., 2016). Although oversights might occur in such large-scale screens, the study implies that Su(z)12 might be a key factor in regulating the temporal dynamics of histone modification in the PG. Recent advances in the Assay for Transposase-Accessible Chromatin with RNA-seq, known as ATAC-seq (Cusanovich et al., 2018), and chromatin immunoprecipitation-RNA-seq technology using samples with a low number of cells (Akhtar et al., 2019) will allow us to examine a genome-wide chromatin modification in the PG in the future.

### How Is the Expression of Halloween Genes Regulated in Other Steroidogenic Tissues Than the Prothoracic Gland?

To date, studies on ecdysteroidogenic TFs have focused on their functions in PG cells. However, ecdysteroid biosynthesis also occurs in other types of cells during embryonic and adult stages.

Studies on *D. melanogaster* and *B. mori* have demonstrated that ecdysteroids are essential for cell differentiation and morphogenesis during embryogenesis (Kozlova and Thummel, 2003; Wang et al., 2018; Fujinaga et al., 2020; Gu et al., 2021; Taira et al., 2021; Yoo et al., 2021). In *D. melanogaster* embryos, ecdysteroids are actively biosynthesized during mid-embryogenesis before the formation of PG primordial cells (Maróy et al., 1988). At this stage, ecdysteroid biosynthesis occurs in the epidermal and amnioserosa cells, in which Halloween genes are strongly expressed (Chávez et al., 2000; Petryk et al., 2003; Niwa et al., 2004, 2010; Warren et al., 2004; Namiki et al., 2005; Ono et al., 2006; Enya et al., 2014). Accordingly, the temporal fluctuation in the expression of Halloween genes during embryogenesis has been shown to correlate with that of the embryonic ecdysteroid titer (Niwa et al., 2010; Enya et al., 2014).

In addition to PG, ovaries are known sites of ecdysteroid biosynthesis. Ovarian ecdysteroids are required for many aspects of oogenesis, such as the regulation of germline stem cell numbers, oocyte maturation, and follicle rupture (Sieber and Spradling, 2015; Uryu et al., 2015; Ables and Drummond-Barbosa, 2017; Knapp and Sun, 2017; Yoshinari et al., 2019; Hoshino and Niwa, 2021) as well as for ovary-gut, and ovary-to-neuron communication (Ahmed et al., 2020; Hadjieconomou et al., 2020; Zipper et al., 2020). Indeed, Halloween genes are expressed in ovarian follicle or nurse cells (Chávez et al., 2000; Petryk et al., 2003; Niwa et al., 2004, 2010; Warren et al., 2004; Namiki et al., 2005; Ono et al., 2006; Domanitskaya et al., 2014; Enya et al., 2014; Ameku and Niwa, 2016; Ameku et al., 2017). However, it is unclear which TFs regulate the ovarian expression of Halloween genes, except for βFtz-f1, which appears to regulate the expression of *dib* in follicle cells (Talamillo et al., 2013).

### Which Transcription Factors Are Essential for the Expression of Halloween Genes in Arthropods Other Than *Drosophila melanogaster*?

As shown in Table 2, most studies on ecdysteroidogenic TFs have been conducted using *D. melanogaster*. Currently, Vvl and FoxO are the exception of an ecdysteroidogenic TF shown to act in other insects. In particular, a knockdown of *vvl* results in defects in ecdysteroid biosynthesis in the *T. castaneum* and *O. fasciatus* coleopteran insects (Cheng et al., 2014; Sarwar et al., 2020). However, besides Vvl, the involvement of other ecdysteroidogenic TFs identified in *D. melanogaster* in the regulation of ecdysteroid biosynthesis has not been explored in other insects. In fact, a comparative gene expression study has reported that, among 13 TFs identified as specifically expressed in the *D. melanogaster* ring glands (Ou et al., 2016), only 2 of their orthologs, *tim* and *vvl*, are expressed in *B. mori* PGs (Moulos et al., 2018). More strikingly, *mld*, *séan*, and *ouib* are only found in *Drosophilidae* species but not in any other insect species (Ono et al., 2006; Komura-Kawa et al., 2015; Uryu et al., 2018). This situation is quite paradoxical, especially for *mld* and *séan*, because *nvd*, the Halloween gene that is transcriptionally induced by the cooperation of Mld and Séan, is widely conserved in most insect species (Yoshiyama et al., 2006; Yoshiyama-Yanagawa et al., 2011; Schumann et al., 2018). These data imply that, during evolution, each phylogenetic class of insects might have acquired or lost essential ecdysteroidogenic TFs that are not conserved in *D. melanogaster*. Therefore, future studies using non-*D. melanogaster* species are extremely important. To search for ecdysteroidogenic TFs in species other than *D. melanogaster*, a large-scale, unbiased RNAi screen system in *T. castaneum* might be useful (Schmitt-Engel et al., 2015; Schultheis et al., 2019). Future challenges with a wide range of insect species will allow us to understand the common and diverse mechanisms of transcriptional regulation of ecdysteroid biosynthesis.

## Author Contributions

TK and RN wrote, revised the manuscript, and approved the submitted version. Both authors contributed to the article and approved the submitted version.

## Conflict of Interest

The authors declare that the research was conducted in the absence of any commercial or financial relationships that could be construed as a potential conflict of interest.

## Publisher’s Note

All claims expressed in this article are solely those of the authors and do not necessarily represent those of their affiliated organizations, or those of the publisher, the editors and the reviewers. Any product that may be evaluated in this article, or claim that may be made by its manufacturer, is not guaranteed or endorsed by the publisher.
